# A Meta-Analysis of Randomized Controlled Trials of Low-Volume Polyethylene Glycol plus Ascorbic Acid versus Standard-Volume Polyethylene Glycol Solution as Bowel Preparations for Colonoscopy

**DOI:** 10.1371/journal.pone.0099092

**Published:** 2014-06-05

**Authors:** Qingsong Xie, Linghui Chen, Fengqing Zhao, Xiaohu Zhou, Pengfei Huang, Lufei Zhang, Dongkai Zhou, Jianfeng Wei, Weilin Wang, Shusen Zheng

**Affiliations:** 1 Division of Hepatobilitary and Pancreatic Surgery, Department of surgery, First affiliated Hospital, School of Medicine, Zhejiang University, Hangzhou, Zhejiang Province, China; 2 Key Laboratory of Combined Multi- Organ Transplantation, Ministry of Public Health, Hangzhou, Zhejiang Province, China; 3 Key laboratory of Organ Transplantation, Zhejiang Province, Hangzhou, Zhejiang Province, China; University Hospital Llandough, United Kingdom

## Abstract

**Background:**

Standard-volume polyethylene glycol (PEG) gut lavage solutions are safe and effective, but they require the consumption of large volumes of fluid. A new lower-volume solution of PEG plus ascorbic acid has been used recently as a preparation for colonoscopy.

**Aim:**

A meta-analysis was performed to compare the performance of low-volume PEG plus ascorbic acid with standard-volume PEG as bowel preparation for colonoscopy.

**Study:**

Electronic and manual searches were performed to identify randomized controlled trials (RCTs) that compared the performance of low-volume PEG plus ascorbic acid with standard-volume PEG as bowel preparation for colonoscopy. After a methodological quality assessment and data extraction, the pooled estimates of bowel preparation efficacy during bowel cleansing, compliance with preparation, willingness to repeat the same preparation, and the side effects were calculated. We calculated pooled estimates of odds ratios (OR) by fixed- and/or random-effects models. We also assessed heterogeneity among studies and the publication bias.

**Results:**

Eleven RCTs were identified for analysis. The pooled OR for preparation efficacy during bowel cleansing and for compliance with preparation for low-volume PEG plus ascorbic acid were 1.08 (95% CI = 0.98–1.28, P = 0.34) and 2.23 (95% CI = 1.67–2.98, P<0.00001), respectively, compared with those for standard-volume PEG. The side effects of vomiting and nausea for low-volume PEG plus ascorbic acid were reduced relative to standard-volume PEG. There was no significant publication bias, according to a funnel plot.

**Conclusions:**

Low-volume PEG plus ascorbic acid gut lavage achieved non-inferior efficacy for bowel cleansing, is more acceptable to patients, and has fewer side effects than standard-volume PEG as a bowel preparation method for colonoscopy.

## Introduction

Colorectal cancer, a major cause of cancer-associated morbidity and mortality, is one of the most common cancers [1]. Colonoscopy has become the standard procedure for the diagnosis of colorectal cancer, and widespread colorectal cancer screening and surveillance have resulted in an increased demand for colonoscopy [2], [3]. Screening is crucial for the early detection and removal of premalignant adenomas or localized cancers in order to reduce morbidity and mortality associated with colorectal cancer [4], [5]. A clean colon without solids or residual brown liquid, which could mask a potential lesion, ensures adequate visualization of the colonic mucosa.

Thus, the level of bowel cleansing, to a large extent, determines the success of colonoscopy [6]. Moreover, the bowel preparation process is the single greatest deterrent to subsequent screening [7]. Some studies had reported that poor bowel preparation is associated with lower rates of adenoma detection, incomplete colonoscopy, and greater procedural technical difficulty [8]–[10]. In particular, inadequate bowel preparation may lead to failure to detect lesions in the right colon [11].

The quality of bowel preparation is dependent on patient compliance, the type of bowel preparation, and the timing of ingestion [12]. Furthermore, for adequate compliance in asymptomatic individuals, an effective screening procedure must ensure high sensitivity and prove to be both safe and tolerable [13].

Sodium phosphate solution (SPS) has been used since the 1990s [14]. Despite the advantage of being more acceptable to patients, SPS may cause greater osmotic effects, drawing plasma water into the gastrointestinal tract [15]. Furthermore, because of its potential risk for clinically significant alterations in serum electrolyte levels and hemodynamic stability, SPS is generally not recommended for patients with renal failure, congestive heart failure, uncontrolled hypertension, or ascites [16]–[19].

An alternative preparation of polyethylene glycol (PEG)-based gut lavage, which was introduced in 1980 [20], is an iso-osmotic solution that passes through the bowel, without absorption or secretion. The standard-volume PEG-based solution has been confirmed as safe and efficacious in colonoscopy [21], even in patients with serum electrolyte imbalances, advanced hepatic dysfunction, acute and chronic renal failure, and congestive heart failure [6]. However, in clinical practice, patients experience problems completing the preparation due to the large volume (4 L) and salty taste of the solution, and they tend to drink less than the full amount, resulting in suboptimal efficacy [22].

Recently, combining ascorbic acid, a laxative, with PEG showed potential to reduce the volume necessary for effective colonic cleansing, while possibly improving tolerance. The absorption of ascorbic acid reaches saturation at high doses [23], [24]. Thus, excess ascorbic acid, which cannot be absorbed, remains in the bowel, where it exerts an osmotic effect, acting synergistically with PEG. The addition of ascorbic acid also appears to improve the taste of the PEG preparation. Therefore, by adding ascorbic acid, the standard volume of PEG (4 L) can be halved to 2 L, and the solution tastes better. Furthermore, some randomized controlled trials have confirmed that this low-volume PEG plus ascorbic acid preparation was as effective as the standard-volume PEG preparation and improved patient satisfaction and compliance [25]–[27].

In the present study, we performed a systematic review and meta-analysis to qualitatively and quantitatively summarize previous high-quality RCTs that compared low-volume PEG plus ascorbic acid with standard-volume PEG preparation in terms of bowel preparation quality. We also sought to statistically summarize secondary outcomes, such as compliance with the preparation, the willingness of the patient to repeat the same bowel preparation, and side effects.

## Methods

### Search Strategy

A computerized search was performed by two independent investigators (W-L.W. and Q-S.X.) in PubMed/Medline, EMBASE, the Cochrane Library, and Google Scholar to identify relevant articles published between 2000 and 2013. We scanned publishers’ databases and conducted manual searches among *surgical endoscopy and other interventional techniques*, *gastrointestinal endoscopy*, *endoscopy*, *digestive endoscopy* and *diseases of the colon and rectum*. The abstracts submitted to the Digestive Disease Week and the ACG national meeting, were also manually searched for accuracy and completeness of data retrieval. The literature search was performed using the following terms: 2L or low-volume polyethylene glycol plus ascorbic acid and 4 L or standard-volume polyethylene glycol and colonoscopy.

### Selection Criteria

Two reviewers (W-L.W. and Q-S.X.) read the titles and abstracts of original articles that compared the performance of low-volume PEG plus ascorbic acid with standard-volume PEG as the preparation method for colonoscopy to select potentially relevant articles. All of the selected articles were collected and reviewed independently by the same reviewers to determine their eligibility for detailed analysis. The inclusion criteria were: (i) randomized controlled trials (RCTs), (ii) adult patients undergoing elective colonoscopy, and (iii) using 2 L PEG plus ascorbic acid and 4 L PEG preparations. Exclusion criteria were duplicate publications (based on the same primary study) and a lack of categorical data on preparation quality or adherence. Review articles, editorials, letters to the editor, and articles enrolling patients younger than 18 years old were also excluded. Disagreements between the two reviewers regarding study selection were resolved by consensus after a face-to-face discussion. If data were missing from a study, the investigator was contacted to provide the missing data if possible. Each study was evaluated by a Jada score [28] and criteria based on Jüni et al [29] to assess the quality of the study.

### Outcomes

The primary outcome was bowel preparation efficacy. This was prespecified as an Ottawa score less than 5, or an excellent or good bowel preparation designation on the Aronchik scale or other non-validated 3-, 4-, or 5-point scales (excellent, good, fair, poor, very poor). As an assessment of bowel preparation tolerability and side effects, a patient’s subjective evaluation of their level of satisfaction and acceptability of the bowel preparation was recorded by studies that administered a periprocedural non-standardized questionnaire to the patient. Data for the secondary outcomes were extracted from the results of these questionnaires. Compliance with bowel preparation was defined as adherence to the bowel preparation prescribed or consumption of at least 75% of the prescribed bowel preparation. The additional secondary outcomes of willingness to repeat the same bowel preparation and side effects, including abdominal cramping/pain, abdominal bloating, vomiting, and nausea, represented affirmative responses to the relevant question from the questionnaires.

### Statistical Analysis

A meta-analysis was performed comparing low-volume PEG plus ascorbic acid solution with standard-volume PEG solution as bowel preparation for colonoscopy by calculating pooled estimates of the quality of the bowel preparation, compliance with the preparation, willingness to repeat the same preparation, and side effects using the odds ratio (OR) with fixed- or random-effects models. Publication bias was assessed using funnel plots. Heterogeneity among studies was assessed by calculating the *I* ^2^ measure of inconsistency, which was considered significant if *I* ^2^>50%. The RevMan 5.2 software from the Cochrane Collaboration was used for the statistical analysis.

## Results

### Study Characteristics

**Figure 1:**
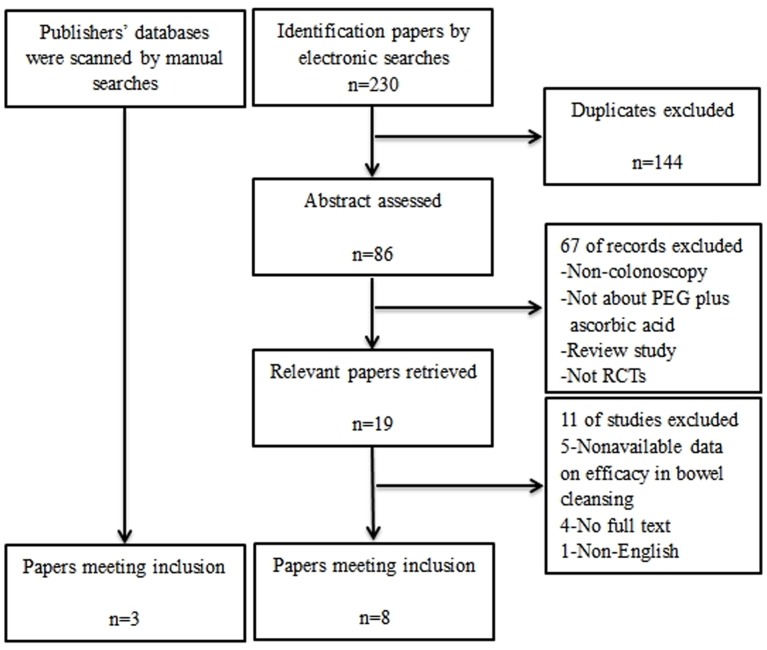
Flowchart of the studies identified for the meta-analysis.

Figure 1 shows the flow of our search results. In total, 230 studies were identified using electronic searches. Excluding duplicates, 86 abstracts were assessed, of which 19 appeared relevant, and the full studies were assessed. Ultimately, eight investigations were identified for inclusion [27], [30]–[36]. And three RCTs [37]–[39] were included by manual searches between publisher’s database and the abstracts submitted to the Digestive Disease Week and the ACG national meeting (Table 1).

**Table 1:** Summary of studies comparing treatment of 2L PEG plus ascorbic acid with 4L PEG solution as bowel preparation for colonoscopy.

Study	Year	Type of study	Blinding	Location	N	Male (%)	Age	Bowel preparation scale	Preparation and dose used	Jadad score
Clark L δ	2007	RCT	Single	NR	294	NR	NR	NR	2L PEG+ascorbic acid VS 4L PEG	1
Ell, C.	2008	RCT	Single	German	308	48.7	58.8±15.4ξ	Non-validated 5-point scale	2L PEG+ascorbic acid VS 4L PEG	3
Lee BC δ	2008	RCT	Single	NR	56	50.0	57.9ξ	NR	2L PEG+ascorbic acid VS 4L PEG	1
Corporaal, S.	2010	RCT	Single	Netherlands	307	48.2	20–87ζ	Non-validated 5-point scale	2L PEG+ascorbic acid VS 4L PEG	2
Marmo, R	2010	RCT	Single	Italy	433	57.5	58.3±14.8ξ	Inverted Ottawa scale	2L PEG+ascorbic acid VS 4L PEG	3
González-Méndez Y δ	2011	RCT	Single	Spain	681	NR	NR	Non-validated 5-point scale	2L PEG+ascorbic acid+Bisacodyl VS 4L PEG+Bisacodyl	1
Pontone, S.	2011	RCT	Single	Italy	142	52.7	20–84ζ	Aronchick scale	2L PEG+ascorbic acid VS 4L PEG+simethicone	3
Jansen, Sita V	2011	RCT	Single	Netherlands	370	41.9	18–92ζ	Non-validated 3-point scale	2L PEG+ascorbic acid VS 4L PEG	2
Valiante, F.	2012	RCT	Single	Italy	332	53	36–85ζ	Aronchick Scale	2L PEG+ascorbic acid VS 4L PEG	3
Gentile, M.	2013	RCT	Single	Italy	120	52.5	20–87ζ	Aronchick scale	2L PEG+ascorbic acid VS 4L PEG+simethicone	3
Ponchon, Thierry	2013	RCT	Single	France	400	53	55.5±12.3ξ	Harefield Cleansing scale	2L PEG+ascorbic acid VS 4L PEG	2

The reference marked by symbol of δ presented in abstract form; ξ values represent mean±standard difference, ζ values represent range of age. PEG, polyethylene.

glycol; RCT, randomized controlled trial; NR, not report.

Table 1 summarizes the characteristics of the eleven studies involving 3431 patients. Regarding bowel preparation scales, non-validated 5-point scales were used in three studies [27], [29], [39], the Aronchick scale in four [31], [33]–[35], the Ottawa scale in one [30], and a non-validated 3-point scale in the final study [32]. Two [31], [34] of the eleven studies compared low-volume PEG plus ascorbic acid with standard-volume PEG plus simethicone as bowel preparations for colonoscopy.

### Primary Outcome: Bowel Preparation Efficacy

**Figure 2:**
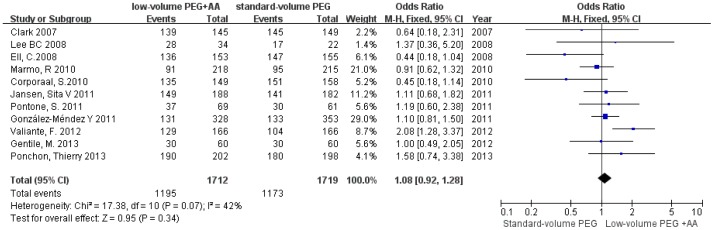
Forest plot showing equal bowel preparation efficacy of low-volume PEG plus ascorbic acid and standard-volume PEG as bowel preparations for colonoscopy.

The forest plot in Figure 2 shows the results of bowel preparation efficacy for the individual studies and for the aggregated studies. Six of the eleven studies reported that low-volume PEG plus ascorbic acid had a higher proportion of patients with excellent or good bowel preparations compared with that of standard-volume PEG. The two studies comparing low-volume PEG plus ascorbic acid with standard-volume PEG plus simethicone showed the same outcome. However, three of the eleven studies found the opposite result. The summary statistic for comparison of low-volume PEG plus ascorbic acid with standard-volume PEG in the eleven studies was an OR of 1.08 (95% CI = 0.92–1.28, P = 0.34, *I* ^2^ = 42%), showing no statistically significant difference, indicating that low-volume PEG plus ascorbic acid solution achieved equal bowel preparation efficacy compared with standard-volume PEG.

### Secondary Outcomes

**Figure 3:**
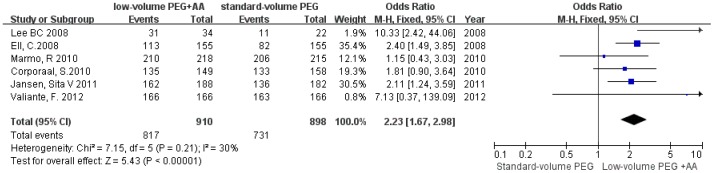
Forest plot depicting better compliance with low-volume PEG plus ascorbic acid than with standard-volume PEG as bowel preparations for colonoscopy.

Six [27], [29], [30], [32], [33], [38] of the eleven studies reported compliance with the preparation, and the pooled OR of compliance with preparation for low-volume PEG plus ascorbic acid was 2.23 (95% CI = 1.67–2.98, P<0.00001, *I* ^2^ = 30%), compared with standard-volume PEG (Fig. 3), suggesting that low-volume PEG plus ascorbic acid had significantly better compliance than did standard-volume PEG, without heterogeneity.

**Figure 4:**
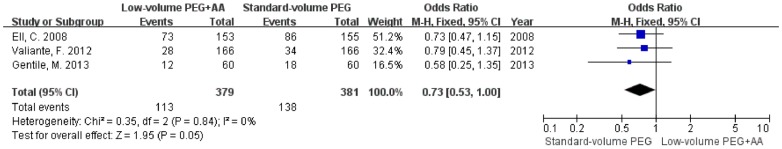
Forest plot revealing fewer overall adverse events with low-volume PEG plus ascorbic acid than with standard-volume PEG as bowel preparations for colonoscopy.

**Figure 5:**
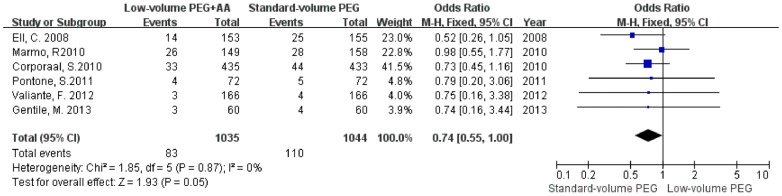
Forest plot showing less vomiting with low-volume PEG plus ascorbic acid than with standard-volume PEG as bowel preparations for colonoscopy.

**Figure 6:**
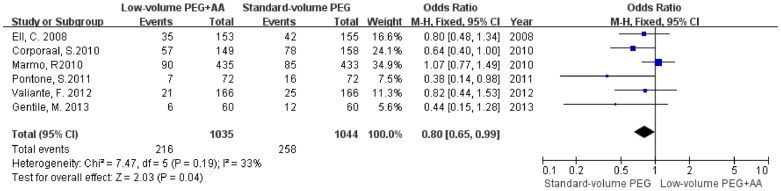
Forest plot showing less nausea with low-volume PEG plus ascorbic acid than with standard-volume PEG as bowel preparations for colonoscopy.

The pooled OR of overall adverse events, willingness to repeat the same bowel preparation, abdominal cramping/pain, abdominal bloating, vomiting, and nausea were 0.73 (95% CI = 0.53–1, P = 0.05, *I* ^2^ = 0%), 0.82 (95% CI = 0.56–1.19, P = 0.29, *I* ^2^ = 0%), 1.10 (95% CI = 0.83–1.45, P = 0.52, *I* ^2^ = 0%), 1.00 (95% CI = 0.73–1.38, P = 0.98, *I* ^2^ = 0%), 0.74 (95% CI = 0.55–1.00, P = 0.05, *I* ^2^ = 0%), and 0.80 (95% CI = 0.65–0.99, P = 0.04, *I* ^2^ = 33%), compared with standard-volume PEG, respectively (Table 2). Thus, low-volume PEG plus ascorbic acid showed significantly fewer overall adverse events and less vomiting and nausea than did standard-volume PEG (Fig. 4–6).

**Table 2:** Secondary outcomes of low-volume PEG plus ascorbic acid VS standard-volume PEG.

Outcomes	Studies n	Patients N	Pooled OR	95%CI	P	I^2^
Overall adverse events	3	760	0.73	0.53–1.00	0.05	0%
Willingness to repeat	3	571	0.82	0.56–1.19	0.29	0%
Abdominal cramping/pain	7	2449	1.10	0.83–1.45	0.52	0%
Abdominal bloating	3	1483	1.00	0.73–1.38	0.98	0%
Vomiting	6	2079	0.74	0.55–1.00	0.05	0%
Nausea	6	2079	0.80	0.65–0.99	0.04	33%

PEG, polyethylene glycol; OR, odds ratios.

**Figure 7:**
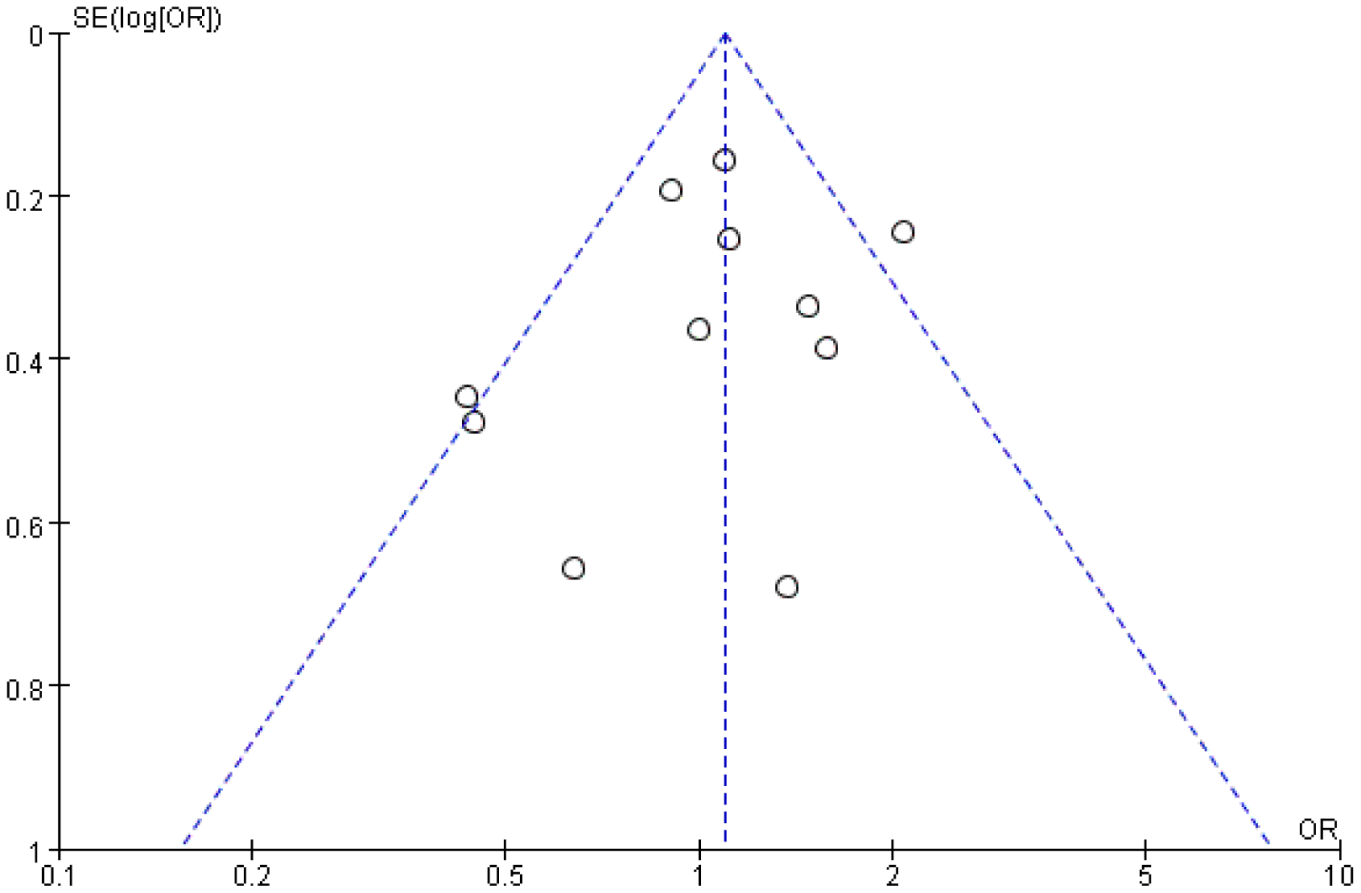
Funnel plot showing no significant publication bias for the primary outcome.

There was no significant publication bias detected for the primary outcome of excellent or good bowel preparation efficacy, as assessed using a funnel plot (Fig. 7).

**Figure 8:**
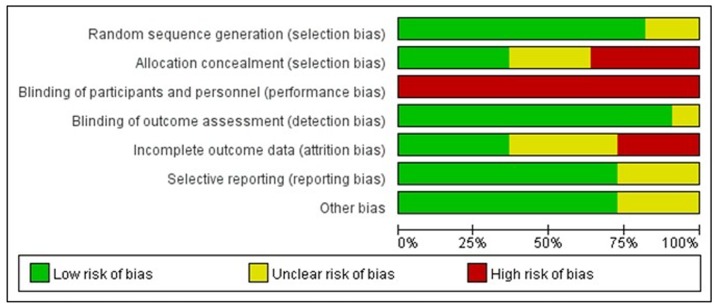
Risk of bias grapy: review author’s judgements about each risk of bias item presented as percentages across all included studies.

And the risk of bias across all included studies was assessed by using the Cochrane Collaboration’s tool (Fig. 8).

## Discussion

Sodium phosphate (SP) has similar bowel preparation efficacy to and better tolerability than that of PEG [40]. However, SP has been associated with renal dysfunction and severe disturbances in electrolyte balance [41]–[44]. In addition, a consensus statement recommends that for children, the elderly, and those with renal insufficiency, bowel preparation should be restricted to PEG-based solutions [45]. Thus, standard-volume (4 L) PEG has been regarded as the gold standard for bowel preparation [46]. However, a difficulty of standard-volume PEG is that many patients are unable or unwilling to consume a 4-L preparation [47], [48].

Because the ideal bowel cleanser is effective, safe for all patient groups, and acceptable to patients, the combined use of low-volume (2 L) PEG and ascorbic acid was recently introduced into clinical practice. However, several investigators have argued that low-volume PEG provides inadequate cleansing of the upper colon and significantly worse bowel preparation than either standard-volume PEG or SP [49]–[51]. Recently, Godfrey [52] reported that there was no significant difference between the two bowel lavage solutions.

Compared to Godfrey’s study, in the present study, we included 2 more RCTs and confirmed by meta-analysis that low-volume PEG plus ascorbic acid solution achieved the same bowel preparation efficacy as did standard-volume PEG (OR = 1.08, P = 0.34, *I* ^2^ = 42%). The two studies comparing low-volume PEG plus ascorbic acid with standard-volume PEG plus simethicone also reported similar bowel preparation quality. This non-inferior efficacy in bowel cleansing benefited substantially from the synergistic osmotic effect achieved with low-volume PEG [23], [24].

Several factors may contribute to heterogeneity among trials. First, variation in timing of bowel preparation may affect preparation quality. The time at which the bowel preparation was started was not uniform among the trials, ranging from 12–48 h before the scheduled procedure. Second, variation in the dosage schedule may also impact bowel preparation efficacy. The dosage schedule included a non-split-dosage schedule, which involved consuming the entire dose the evening prior to the day of the planned colonoscopy, and a split-dosage schedule, which involved drinking half the dose the afternoon prior and the other half the morning of the the procedure. Third, variation in dietary instructions prior to and during the preparation may have contributed to the heterogeneity. Among the trials included, the dietary instructions were not uniform, ranging from a regular diet to a clear liquid diet for lunch and a clear liquid diet in the evening. Fourth, the diverse use of bowel preparation scales potentially led to heterogeneity. We found that the eight studies used differing preparation scales, such as the Aronchick scale, the Ottawa scale, and non-validated 3-, 4-, and 5-point scales. Finally, the use of different adjuvants probably resulted in heterogeneity. Two studies [32], [35] used ascorbic acid and simethicone, but the other studies used ascorbic acid only.

Colonoscopy is increasingly being used to screen healthy patients (or those with relatively minor symptoms) for bowel cancer [53]. Inability to consume the complete preparation may reduce screening efficacy [49]. Physicians favor preparations associated with best patient compliance to achieve optimal results. Patients favor preparations that are low in volume, palatable, and in easy-to-complete regimens [45]. Given its desirability to physicians and patients, a new low-volume preparation has been developed using ascorbic acid added to a 2-L PEG solution. Godfrey’s study [52] presented a very valuable data on satisfactory bowel preparation of the two bowel lavage solution, however, it was a pity for no investigation of compliance with the two bowel lavage solutions. The present study confirmed that low-volume PEG plus ascorbic acid has significantly better compliance than standard-volume PEG without heterogeneity (OR = 2.23, P<0.00001, *I* ^2^ = 30%). The individual studies [27], [29], [30], [32], [33] included in the analysis consistently reported that a larger proportion of patients in the low-volume preparation group consumed at least 75% of the prescribed bowel preparation than did patients enrolled in the standard-volume preparation group. The better compliance may result from the consumption of a smaller volume of liquid and the more palatable ascorbic acid. Better compliance, combined with the laxative effect of ascorbic acid, may account for the similar bowel preparation efficacies between the lower- and standard-volume preparations. The low-volume preparation was especially applicable to outpatients who conducted their intake independently.

In the present study, we also compared overall adverse events, abdominal cramping/pain and bloating, vomiting, and nausea between the low-volume PEG plus ascorbic acid and the standard-volume PEG groups. We found that there was no significant difference in abdominal cramping/pain or bloating between the groups. However, we did see that the low-volume group had significantly fewer overall adverse events and less vomiting and nausea than did the standard-volume group. This significant difference may result from the safety of ascorbic acid, even at high doses [54], [55], and the lower volume of PEG, reducing any PEG-based electrolyte or volume alterations [34].

In summary, optimal bowel cleansing should be not only effective but also safe for all patients. With non-inferior bowel preparation efficacy and the advantages of fewer adverse events and better compliance, the low-volume PEG plus ascorbic acid solution was clearly more applicable to bowel preparation for colonoscopy.

## Supporting Information

Checklist S1 **PRISMA checklist.**
(DOC)
